# Separation of Lead with a Novel Ion Separating Agent Prepared by Clothing a Chitin Whisker on a Potassium Tetratitanate Whisker

**DOI:** 10.3390/ma10030262

**Published:** 2017-03-06

**Authors:** Juan Liu, Qin-guo Li, Wen-jing Xue

**Affiliations:** College of Environment and Safety Engineering, Qingdao University of Science and Technology, Qingdao 266042, China; m13730918131@163.com (Q.-g.L.); xwjwenjing@163.com (W.-j.X.)

**Keywords:** ion separating, chitin whisker, potassium tetratitanate whisker, coating

## Abstract

Separation of Pb^2+^ from Cu^2+^-Pb^2+^ mixed solution by a newly-developed ion separating agent was examined, which was obtained by clothing chitin whiskers (ChW) on the surface of potassium tetratitanate whiskers (PTW). The separation capability and mechanism of the ion separating agent (ChW-PTW) was determined, based on the difference of the adsorption isotherm pattern and the adsorption kinetics model between ChW and PTW on Cu^2+^ and Pb^2+^, respectively. The results showed that the adsorption process of ChW could be described by Freundlish isotherm. The adsorption affinity of Cu^2+^ (k_F_ = 0.085·g^−1^) on ChW was greater than Pb^2+^ (k_F_ = 0.077 g^−1^). The adsorption pattern of PTW was inclined to the Langmuir isotherm, and Pb^2+^ (k_L_ = 310.59 L·mmol^−1^) could be obviously more easily adsorbed on PTW than Cu^2+^ (k_L_ = 25.85 L·mmol^−1^). The experimental data both fitted well with the pseudo-second order kinetics. The reaction rate of Pb^2+^ (k_2_ = 4.442 for ChW and k_2_ = 0.846 for PTW) was greater than that of Cu^2+^ on both ChW and PTW, while the diffusion rate of intra-particles of PTW was much higher than ChW. The adsorption model of ChW and PTW could illustrate well the separation mechanism of ChW-PTW and allowed for relevant results.

## 1. Introduction

Separation and recovery of heavy mental ions have always been a significant topic because they are non renewable. There have been some technologies for ions removal from wastewater, such as chemical precipitation, membrane filtration, electrochemistry, adsorption, and so on, in which adsorption has generally been used as an attractive method for industry wastewater treatment. Chen [1] prepared a kind of adsorbent based on copper hexacyanoferrate, which were chemically deposited on the electrode as a film. Metal ions could be adsorbed on the film under the electric power. Li [2] synthesized Cd(II) ion-imprinted polymer with imprinting technology by using allylthiourea as a functional monomer and cadmium chloride as the template. It has higher selectivity for the separation of Cd(II) ions from solutions. However, obvious disadvantages appeared with respect to these methods, such as larger energy consumption, complex processing, and giving rise to the transfer of metal ions into water in the process of preparation and use. Thus, it was necessary to develop a novel adsorbent which was low cost, environmental friendly, simple to prepare, and have good performance for separating metal ions.

Potassium tetratitanate whiskers (PTW) are a kind of crystal with a chainlike and open-stratified structure. It has attracted more attention because it is environmentally friendly, non-toxic, and has strong mechanical properties. The interlayer potassium ions can play an important role in ion exchange with other positive ions, such as Pb^2+^, Cr^2+^, Cu^2+^, and so on [3,4,5,6]. Additionally, PTW has powerful reactivity to be surface modified. Ce^3+^-imprinted functionalized PTW sorbent was prepared using surface imprinted technology [7] and the surface imprinted polymer composites (MIP/K_2_Ti_4_O_9_) were prepared using dibenzothiophene (DBT) as the template, 4-vinylpyridine as the functional monomer, and potassium tetratitanate whiskers as the carrier [8]; Ogawa et al. intercalated alkylmmonium cations into the layer of titanate [9,10,11,12]. These reports all illustrated that surface modification could promote the ion exchange properties of PTW.

Chitin is a building block of the skeletons of crustaceans, insects, and diatoms. Chitin/chitosan-based materials have been widely applied as adsorbents for heavy metal ions or radioactive elements from wastewater [13,14,15,16]. The high adsorption property of chitin/chitosan-based materials could be attributed to: (i) high hydrophilicity due to the large number of hydroxyl groups of glucose units; (ii) the presence of a large number of functional groups; (iii) high chemical reactivity of these groups; and (iv) the flexible structure of the polymer chain [17]. Gomes [18] studied the interaction between metal cations and chitin/chitosan by means of density functional theory. For chitin, compounds with Cu^2+^ were more stable than others, and Cu^2+^ preferred to combine with the oxygen atom of the amide group than the nitrogen atom of the amide group. Wysokowski [19] proposed in their review that chitin-based inorganic-organic materials could be obtained under hydrothermal conditions, such as the formation of chitin/SiO_2,_ chitin/ZrO_2_, and chitin/ZnO due to hydrogen bonds. Chitin whiskers (ChW) are widely applied as reinforcing agents because of its nontoxicity, biodegradability, and excellent mechanical behavior [20,21], can be obtained by removing the amorphous domains of chitin through acid hydrolysis [22,23,24,25]. Meanwhile, ChW has similar reactivity and adsorbability with chitin because they both have plenty of hydroxyl groups and acetyl amino groups [20,26,27,28,29]. For example, Kalaprasad [30] described the surface chemical modification of hydroxyl on ChW with various reagents to improve applied properties. ChW showed higher adsorption capability for crystal violet compared to other adsorbents [31]. Blending products of ChW and layered rectorite could enhance physicochemical properties of chitosan film due to cooperation effects. Moreover chitin hydrogel could be reinforced with TiO_2_ nanoparticles to enhance the adsorption of arsenic [32]. 

To sum up, it is feasible for ChW to combine with PTW as a novel composite material. According to the earlier experimental data, adsorption capacities of PTW for metal ions are much larger than that of ChW. When ChW film is attached to the surface of the PTW, ChW film outside will be like a barrier to block other impurity ions, and pure Pb^2+^ will enter inside and be adsorbed by PTW. There is almost no literature referring to ion separation by such an organic-inorganic compound adsorbent.

The purpose of the present study was to discuss the separation property and mechanism of the novel ion separating agent, named ChW-PTW, prepared by clothing ChW on the surface of PTW under heating treatment. Lead is one of the most toxic heavy metals and is used in industry widely. It is exhausted quickly with economic development. Thus, it was significant to choose Pb^2+^ as the subject. Firstly, a batch of adsorption experiments of ChW and PTW on Pb^2+^ and Cu^2+^ were conducted to determine the adsorption isotherm pattern and adsorption kinetics model, on which adsorption affinity, adsorption rate constant, and diffusion rate constant could be estimated. Then the separation capability and mechanism of ChW-PTW for Pb^2+^ from Cu^2+^-Pb^2+^ mixed solution was discussed based on the difference of adsorption processes between ChW and PTW on Cu^2+^ or Pb^2+^.

## 2. Experiments

### 2.1. Materials

Chitin whiskers (ChW) were prepared by a two-step acid-alkali process [13]. Potassium tetratitanate whiskers (PTW) were purchased from Shanghai Dian Yang Industry Co., Ltd. (Shanghai, China). Lead nitrate and cupric nitrate were obtained both from Tianjin Bodi Chemical Limited by Share Ltd. (Tianjing, China).

### 2.2. Preparation of ChW-PTW

#### 2.2.1. Preparation of ChW

A certain amount of chitin powder was added into 3 N HCl with the material ratio of 1:30 (*g*:*v*). The sample was boiled and stirred for 1.5 h to hydrolyze chitin to obtain ChW. Then the mixture of ChW and residual chitin was centrifuged. The milky supernatant was collected and the pH adjusted with sodium hydroxide. Thus, colloidal ChW was obtained. The detailed preparation procedure was described in our previous paper [13].

#### 2.2.2. Preparation of ChW-PTW

A certain amount of PTW was mixed with the emulsion of ChW by stirring under 500 r/min for 1 h. Then the free water in the sample was evaporated under stirring at 300 r/min at 100 °C. After cooling to ambient temperature, the sample was vacuum dried at −0.09 Mpa and 105 °C. The product was added to the emulsion of ChW again, and the above procedures were repeated seven times. The final product was obtained with ChW film clothing on the surface of PTW (ChW-PTW). The product was immersed in the cetyl sodium sulfate liquor with pH = 5–6 for a certain time to remove residual ChW. The product could be used after washing and drying.

### 2.3. Characterization

Scanning electron microscope (SEM) images were taken on a JEOL JSM-6700F SEM instrument (Japan Electron Optics Laboratory Co. Ltd., Tokyo, Japan), the testing voltage was 12 KV, and the magnification was 2000–50,000 times.

The concentration of Cu^2+^ and Pb^2+^ was determined by TAS-986 (Beijing Purkinje General Instrument Co., Ltd., Beijing, China) under room temperature.

### 2.4. Difference of Adsorption Property between ChW and PTW

The difference of the equilibrium adsorption isotherm and adsorption kinetics between ChW and PTW was studied to analyze the separation mechanism of ChW-PTW for Pb^2+^ from Cu^2+^-Pb^2+^ mixed solution. The absorption process can be expressed by formulas, as follows:

2Chi- + M^2+^ ⇌ M(Chi)_2_ (1)

K_2_O·(TiO_2_)_4_ + M^2+^ ⇌ MO·(TiO_2_)_4_ + 2K^+^ (2)

where Chi- are activated adsorption sites on the surface of ChW; M^2+^ represents metal ions.

#### 2.4.1. Adsorption Isotherm

The equilibrium adsorption isotherm tests were conducted by transferring 0.10 g adsorbents and 20 mL Cu^2+^ or Pb^2+^ solution (0.15–2.5 mmol/L) to triangular flasks in a temperature-controlled orbital shaker (150 rpm). Experiments were performed at 25 °C for 48 h. 

Langmuir, Freundlish, and Temkin equations (Equations (3)–(5), respectively) were used to estimate the isotherm parameters for ChW/PTW on Cu^2+^ or Pb^2+^.

(3) $$q_{e}=\frac{q_{m}\cdot bC_{e}}{1+bC_{e}}$$

(4) $$q_{e}=KC_{e}^{n}$$

(5) $$q_{e}=\frac{RT}{b_{T}}\ln\left(A_{T}C_{e}\right)$$

where *q_e_* and *q_m_* are the equilibrium and maximum monolayer adsorption capacities (mmol/g), respectively; *C_e_* is the equilibrium concentration of mental ions in the solution (mmol/L); *b* is the Langmuir adsorption equilibrium constant that is related to the binding energy (L/mg); *K* is the Freundlich constant that is related to the adsorption capacity (mmol/g); and *n* is the adsorption intensity parameter.

#### 2.4.2. Adsorption Kinetics

Adsorption kinetics experiments were carried out by blending 0.10 g adsorbent with 20 mL Cu^2+^ or Pb^2+^ solution (0.94 mmol/L). The samples were shaken at 25 °C for 10 min, 20 min, 40 min, 60 min, 90 min, 180 min, and 360 min, respectively.

Pseudo-first-order (Equation (6)) and pseudo-second-order (Equation (7)) equations were used to discuss the adsorption rate constant of Cu^2+^ or Pb^2+^ on ChW/PTW. Webber-Morris model (Equation (8)) was used to study intra-particle diffusion model, as follows:

(6) $$q=q_{e}\left(1-e^{-k_{1}t}\right)$$

(7) $$q=\frac{k_{2}q_{e}^{2}t}{1+k_{2}q_{e}t}$$

(8) $$q=k_{ind}t^{0.5}+C$$

where *q* is the adsorption quantity at time *t* (mmol/g); *t* is the contact time (min); k_1_, k_2_ are reaction rate constant of pseudo-first-order model (min^−1^) and pseudo-second-order model (g/mg·min), respectively; *k_ind_* is intra-particle diffusion rate constant (mmol/g·min^0.5^); and *C* is the constant related to the thickness of the boundary layer (mmol/g).

### 2.5. Separation of Double-Ions-Mixed Solution

#### 2.5.1. Ionic Adsorption

The separation experiments were operated by immersed 0.10 g ChW-PTW into 20 mL of the mixed solution with 1.888 mmol/L of Cu^2+^ and 0.944 mmol/L of Pb^2+^, respectively. Then the samples were oscillated under 25 °C for a certain time. Monitoring the concentration of Cu^2+^ and Pb^2+^ in the supernatant to estimate the adsorption quantity of ChW-PTW. 

#### 2.5.2. Determination of Separation Capability

ChW-PTW, which had adsorbed the mixed ions in the adsorption process, was collected and dried at 80 °C, then transferred into HNO_3_ (pH = 1) and vibrated at 25 °C for 12 h to peel off ChW from the surface of PTW. ChW would enter into the supernatant to form an emulsion while PTW settled to the bottom. The emulsion and the sediment were separated, purified, and dried to obtain ChW and PTW, respectively. ChW and PTW were immersed into 5 mol/L HNO_3_ alone for 24 h to desorb Cu^2+^ and Pb^2+^. The concentration of Cu^2+^and Pb^2+^ in both solutions was detected to assess the adsorption quantity of the ChW film and PTW layer, and then the separation ability of the sorbent was estimated. 

## 3. Results and Discussions

### 3.1. Morphological and Structural Characteristics

**Figure 1:**
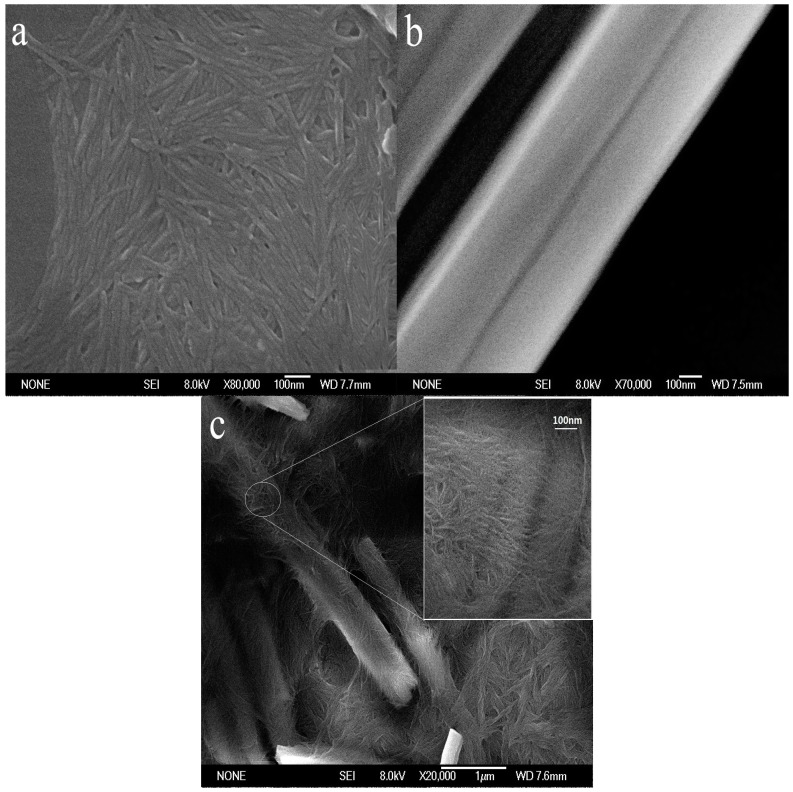
The SEM of (**a**) ChW; (**b**) PTW; and (**c**) ChW-PTW.

The morphology of ChW, PTW, and ChW-PTW are shown in the Figure 1. It can be revealed in Figure 1a that the length of the ChW is about 80 nm to 300 nm. It is well distributed and takes on a membrane shape. It can be seen from the Figure 1b that PTW has a smoother and larger surface than ChW, and it presents stiff rod-like. In the Figure 1c, it shows the morphology of ChW-PTW from an overall perspective and a partially enlarged view. Obvious coalescence between ChW and PTW occurs. ChW is even-distributed on the surface of PTW and clads PTW as a film.

ChW has enough activity binding sites, such as hydroxyl, acetyl, and amidogen, and there is powerful electronegativity on the surface of PTW due to Ti-O. When PTW was mixed with ChW emulsion completely and operated by heating treatment, a new composite material was obtained by hydrogen bonding between ChW and PTW.

### 3.2. The Adsorption Property of ChW and PTW

The optimal adsorption conditions were determined, when 0.1 g of adsorbent was added into 20 mL solution with a concentration of 1.0 mmol/L, optimal pH of the solution was 5–6, the suitable temperature was 25 °C, and the time needed to reach equilibrium was 2–3 h. At this time, the adsorption capacities and comparison with other adsorbents were listed in Table 1.

It can be seen from Table 1 that adsorption capacities of PTW for Cu^2+^ and Pb^2+^ are both much larger than that of ChW. This difference will contribute to the ion separation. To analyze the separation process of the composite, it is necessary to research each removal and diffusion pattern of ChW and PTW.

Compared with other adsorbents, the adsorption capacities are lower, possibly because that: (1) adsorbability of ChW itself is weaker than chitin; (2) before preparing of the composite material, ChW and PTW were both unground. The composite material was also unground, because the structure of the composite was very important to assure the separation degree. As contrast samples, ChW and PTW were unground when batch of experiments were carried out for isotherm and kinetics.

The symbols used in adsorption isotherm and kinetics were listed in Table 2.

#### 3.2.1. Adsorption Isotherm

**Figure 2:**
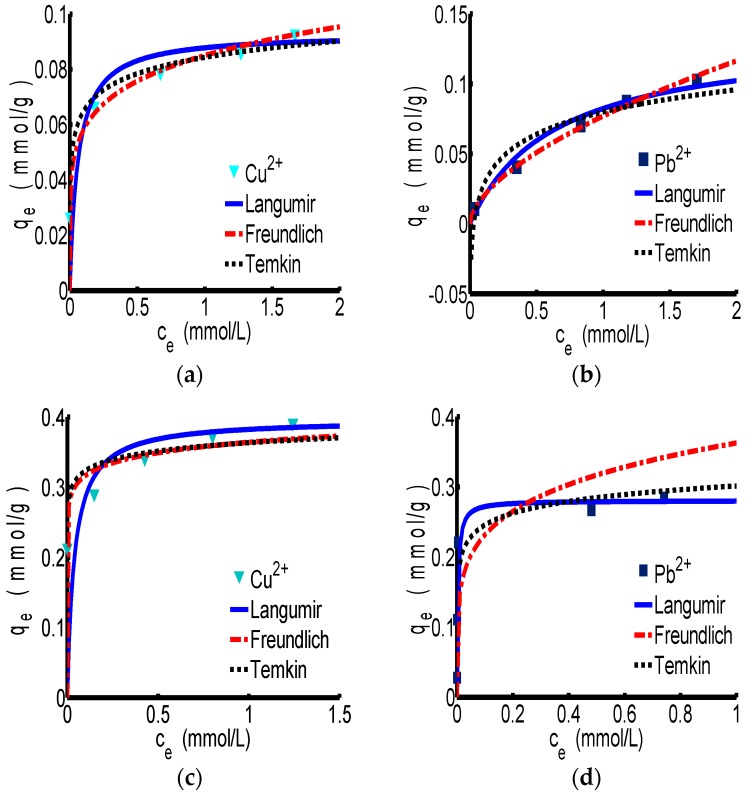
The adsorption isotherm of ChW and PTW. (**a**) ChW-Cu^2+^; (**b**) ChW-Pb^2+^; (**c**) PTW-Cu^2+^; and (**d**) PTW-Pb^2+^.

The adsorption isotherm curves of Langmuir, Freundlich and Temkin for ChW and PTW are revealed in Figure 2. Figure 2a,b correspond to the adsorption process of ChW on Cu^2+^ and Pb^2+^, respectively. According to the parameters in Table 3, the correlation coefficient (R^2^) of the Freundlich model for ChW is 0.998 for Cu^2+^ and 0.999 for Pb^2+^, which is slightly larger than that of Langmuir and Temkin, indicating that the absorption pattern of ChW for Cu^2+^ and Pb^2+^ is more inclined to the Freundlich adsorption model. In this case, k_F_ was 0.085 g^−1^ for Cu^2+^ and 0.077 g^−1^ for Pb^2+^, that is to say, binding activity of Cu^2+^ on ChW is stronger than that of Pb^2+^.

The absorption pattern of PTW on Cu^2+^ and Pb^2+^ fits well to the Langmuir model, as shown in the Figure 2c,d and Table 3. At this time, the k_L_ was 25.85 L·mmol^−1^ for Cu^2+^ and 310.59 L·mmol^−1^ for Pb^2+^, indicating that Pb^2+^ can be captured by PTW much more firmly than Cu^2+^.

The adsorption pattern of ChW on Cu^2+^ and Pb^2+^ is in accordance with the Freundlich isotherm, and Cu^2+^ will be adsorbed more easily than Pb^2+^. The adsorption pattern of PTW on Cu^2+^ and Pb^2+^ can be represented by the Langmuir isotherm, and the adsorption of Pb^2+^ was much stronger than that of Cu^2+^.

#### 3.2.2. Adsorption Kinetics

**Figure 3:**
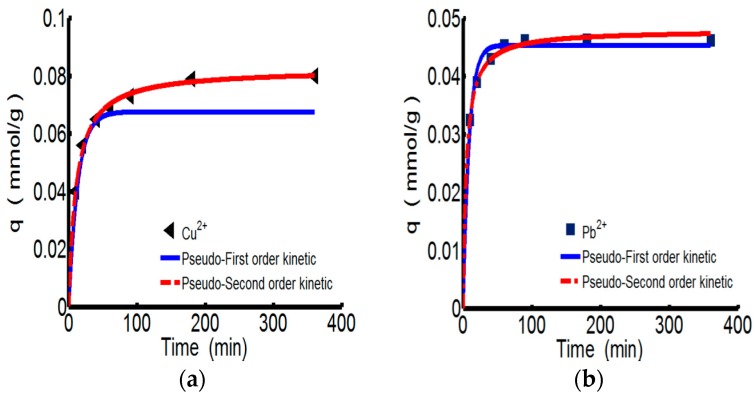

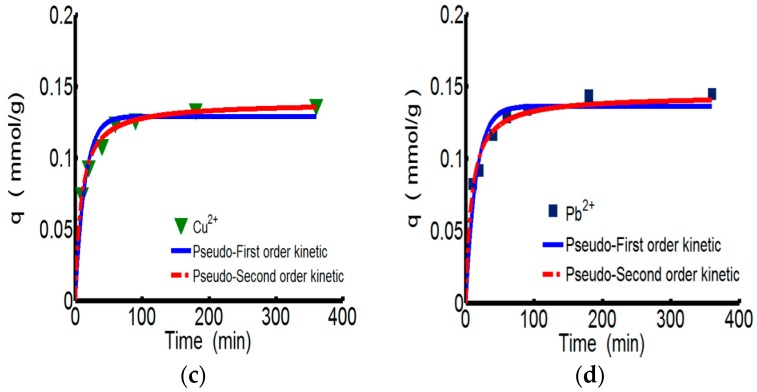
The kinetics curves of ChW and PTW. (**a**) ChW-Cu^2+^; (**b**) ChW-Pb^2+^; (**c**) PTW-Cu^2+^; and (**d**) PTW-Pb^2+^.

Figure 3 shows the adsorption kinetics curves of ChW or PTW on Cu^2+^ and Pb^2+^. Relevant parameters for pseudo-first-order and types 1–4 of pseudo-second-order were shown in the Table 4. All of the models fit the experiment data very well. However considering the conformity of data and the calculated values expressed by the correlation coefficient (R^2^), the pseudo-second-order kinetics are more suitable for describing the adsorption behaviors of both ChW and PTW. The results illustrate that ions can combine with active sites on ChW or PTW by covalent chemical bonds [2].

As for the pseudo-second-order kinetics, k_2_ and *q_e_* can be calculated from the plots for the linear forms of pseudo-second-order kinetics models of types 1–4 [34] given in Table 5. The values of k_2_, *q_e_*, *h*, and *q*_e, cal_ obtained from the four linear forms of pseudo-second-order equations were found to be different. Giving consideration to both correlation coefficients and differences between the experimental (*q*_e, exp_) and calculated sorption capacities (*q*_e, cal_), the adsorption of ChW based on Cu^2+^ and Pb^2+^ better fits type 1, while adsorption of PTW can be described by type 2. Pseudo-second-order models of types 3–4, although the high values of their correlation coefficients, cannot be taken into consideration because of the larger differences between the experimental and calculated sorption capacities.

The reaction rate constant (k_2_) of ChW on Cu^2+^ is 1.152, much lower than that of Pb^2+^ (6.518), while k_2_ of PTW on Cu^2+^ is 0.825, slightly smaller than that of Pb^2+^ (0.846). Demonstrating that Pb^2+^ can be more rapidly captured on both available adsorption sites of ChW and PTW than the adsorption of Cu^2+^.

Weber-Morris model is applied to determine the rate-limiting step in this adsorption system, which represents the time dependent intra-particle diffusion of the solvend. If the Weber-Morris curve of *q* (mmol/g) against *t*^0.5^ (min^0.5^) gives a straight line, the adsorption process is diffusion controlled. If the plot is multi-linear and the lines do not pass through the origin, then a combination of two or more processes influence the adsorption [36].

**Figure 4:**
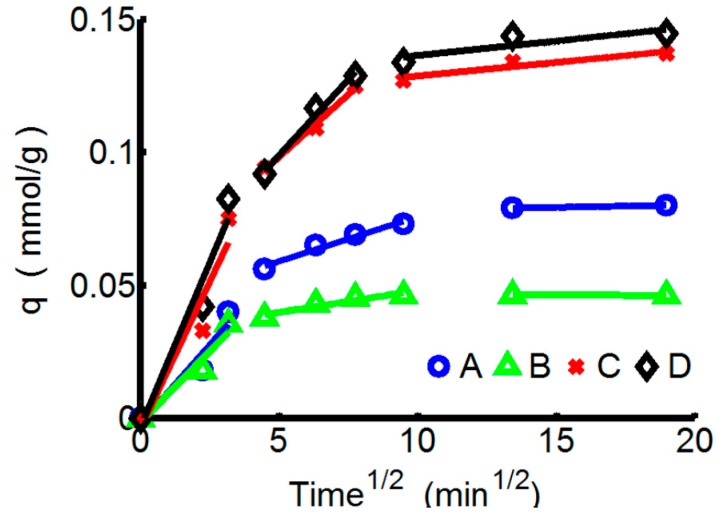
The kinetics curves of ChW and PTW based on the Weber-Morris pattern. (**A**) ChW-Cu^2+^; (**B**) ChW-Pb^2+^; (**C**) PTW-Cu^2+^; and (**D**) PTW-Pb^2+^.

Multi-linear relationships that have three different linear regions with different slopes are shown in Figure 4. The intra-particle diffusion rate constants (k_ind1_, k_ind2_, k_ind3_) correspond with the diffusion rates which can be calculated from the slopes of the linear curves and changes in different adsorption stages. The diffusion rate constants are presented in Table 6, along with the correlation coefficients. The initial region (0–10 min) is the sharpest and corresponds to the external adsorbate diffusion in the boundary layer. That is, ions could quickly diffuse to exposed adsorption sites on the surface, and k_ind1_ of Pb^2+^ towards PTW is significantly higher than that of Pb^2+^ towards ChW. The second region (20–90 min) relates to the gradual adsorption stage in which the intra-particle diffusion is potentially the rate-limiting step. When the external surface is nearly saturated, the ions gradually pass through the surface pores and will be retained in the micropores of the particles. At this stage, the diffusion resistance increases and the diffusion rate decreases. k_ind2_ of ChW-Pb^2+^ decreases from 0.0106 mmol/g·min^0.5^ to 0.0016 mmol/g·min^0.5^, reduces to about 15%. In contrast, k_ind2_ of PTW-Pb^2+^ decreases from 0.0247 mmol/g·min^0.5^ to 0.0114 mmol/g·min^0.5^, reduces to about 46%, illustrating that after 20 min, the diffusion resistance of Pb^2+^ on ChW will be far greater than on PTW. The third region (>90 min) is a plateau that represents the final equilibrium stage, k_ind3_ especially of the adsorption ChW reduce to nearly zero. By contrast, PTW can absorb ions continuously at this stage (k_ind3_ was 0.001 mmol/g·min^0.5^ for both Cu^2+^ and Pb^2+^).

### 3.3. The Separation Effect of Double-Ion-Mixed Solution

**Figure 5:**
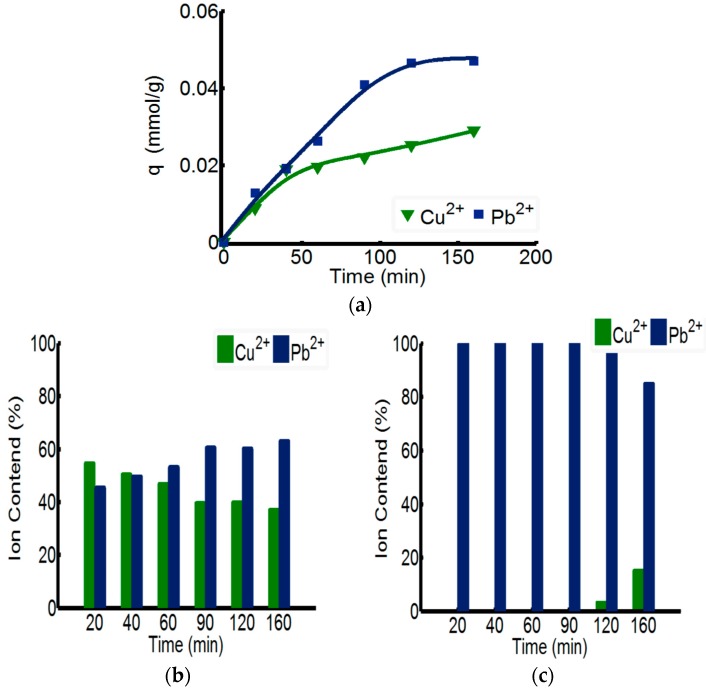
The adsorption capacity of ChW-PTW among Cu^2+^ and Pb^2+^ in a double-ion-mixed solution. (**a**) Adsorbing capacity of ChW-PTW with time; (**b**) the percentages of Cu^2+^ and Pb^2+^ in ChW film; and (**c**) the percentages of Cu^2+^ and Pb^2+^ in the PTW layer.

The separating capability of ChW-PTW on Pb^2+^ from Cu^2+^-Pb^2+^ mixed solution is discussed in Figure 5. As shown in the Figure 5a, the overall adsorption rate of ChW-PTW on Pb^2+^ is quicker than that of Cu^2+^. When the time reaches 160 min, the adsorbance of Pb^2+^ can be up to 0.046 mmol/g, more than that of Cu^2+^. This outcome can be explained by the test data of adsorption kinetics, as the adsorption rate constant of Pb^2+^ on ChW or PTW is both larger than that of Cu^2+^.

The relative percentage quantity of Cu^2+^ and Pb^2+^ in the ChW film on the surface of PTW is shown in Figure 5b. The relative mass percent of Cu^2+^ gradually reduces as time progresses, while that of Pb^2+^ gradually increases. Figure 5c shows the relative percentage quantity of Cu^2+^ and Pb^2+^ in the inner PTW layer. When adsorbed within 90 min, the mass percent of Pb^2+^ was 100%, but when the time was prolonged to 120 min, Cu^2+^ could pass through the ChW film and permeate into the PTW layer. 

In summary, Pb^2+^ can be adsorbed more quickly on ChW-PTW than Cu^2+^ and is more inclined to be combined on the PTW layer. When adsorbing within 90 min, ions on the PTW are pure Pb^2+^. Thus, ChW-PTW will be a promising ion separating agent, and can play an important role to purify Pb^2+^.

### 3.4. The Possible Separation Mechanism of ChW-PTW

Based on the isotherm patten and kinetics model of ChW and PTW, (1) the reaction rate of Pb^2+^ is greater than that of Cu^2+^ on both ChW and PTW; (2) the adsorption affinity between Cu^2+^ and ChW is much larger than that of PTW; (3) PTW has greater advantage to adsorb Pb^2+^; (4) the diffusion resistance of Pb^2+^ in the intra-particle of PTW is much smaller than ChW in the second stage; and (5) the third stage begins after adsorbing 90–120 min. At this time, adsorption of Cu^2+^ on ChW is over, and Cu^2+^ can pass through the ChW film and permeate into the PTW layer. 

**Figure 6:**
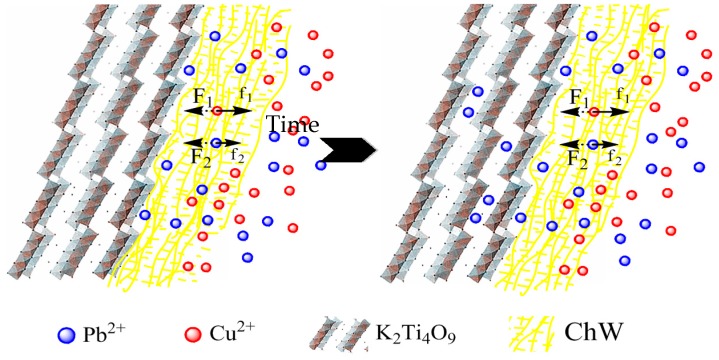
The separation process and mechanism of the double-ion-mixed solution.

When Cu^2+^ and Pb^2+^ contacted with the composite material ChW-PTW, the ChW film could catch Cu^2+^ firmly and block Cu^2+^ from entering into the PTW layer. Most of the chelation sites of ChW could be occupied by Cu^2+^. On the other hand, Pb^2+^ would adsorb on the ChW layer prior to Cu^2+^, but the adsorption capability (k_F_) and diffusion rate (k_ind2_) of Pb^2+^ on ChW was less than Cu^2+^, moreover, the diffusion rate (k_ind2_) of Pb^2+^ on PTW was much larger than on ChW. Then partial Pb^2+^ chelated on the residual active sites of external ChW film quickly, other Pb^2+^ would continuously transfer into the interior-ChW-PTW and adsorbed on the PTW layer by ion exchange. The possible separation process and mechanism of the double-ion-mixed solution is revealed in the Figure 6.

## 4. Conclusions

A newly-developed ion-separating agent was prepared by clothing ChW on the surface of PTW. The microstructure of the product (ChW-PTW) was observed by scanning electron microscope (SEM). The separation capability and mechanism of ChW-PTW on Pb^2+^ from mixed solution was discussed on the foundation of the difference of the adsorption isotherm pattern and adsorption kinetics model between ChW and PTW on Cu^2+^ and Pb^2+^, respectively.

The results show that: (1) ChW can form a kind of compact film, and is well-distributed on the surface of PTW; (2) the reaction rate of Pb^2+^ is greater than that of Cu^2+^ on both ChW and PTW, while the binding affinity between Cu^2+^ and ChW is larger, and PTW has greater advantage to adsorb Pb^2+^; (3) the different reaction rate and binding affinity could explain the separation mechanism perfectly; (4) the intra-particle diffusion resistance of Pb^2+^ on PTW is much smaller than on ChW; and (5) the ion separating agent prepared by ChW and PTW was promising to remove Pb^2+^ from Cu^2+^-Pb^2+^ mixed solutions. There were pure Pb^2+^ on the inner PTW layer when adsorbed within 90 min. This material could successful employed in water pollution control or in purifying of Pb^2+^ ions from industry wastewater.

## Figures and Tables

**Table 1:** The adsorption capacities of ChW, PTW, and a comparison with other adsorbents.

Samples	Adsorption Capacities mmol/g	Reference
ChW-Cu	0.080	-
ChW-Pb	0.075	-
PTW-Cu	0.362	-
PTW-Pb	0.296	-
Mg_2_Al–LS–LDH composite-Cu	1.000	[33]
Mg_2_Al–LS–LDH composite-Pb	0.594	[33]
Peat-Pb	0.398	[34]
Lignin/inorganic oxide system-Cu	1.312	[35]

**Table 2:** Symbol descriptions.

Symbol	Description	Unit
k_L_	The adsorption affinity	L·mmol^−1^
k_F_	Characterization of adsorption capacity	g^−1^
A_t_	The maximum adsorption equilibrium constant	L·mmol^−1^
*q_e_*	Adsorption capacity	mmol·g^−1^
k_1_	Reaction rate constant for pseudo-first-order kinetic	min^−1^
k_2_	Reaction rate constant for pseudo-second-order kinetic	g·min·mmol^−1^
R_2_	Correlation coefficient transformation	-
E^2^	The estimate of the error variance	-

**Table 3:** The parameter of the adsorption isotherm.

Isotherm Type	Parameter	ChW		PTW	
		Cu^2+^	Pb^2+^	Cu^2+^	Pb^2+^
Langmuir	k_L_	16.725	1.574	25.851	310.59
	R^2^	0.994	0.943	0.997	0.999
	E^2^	4.65 × 10^−4^	1.93	6.90 × 10^−3^	3.00 × 10^−3^
Freundlich	k_F_	0.085	0.077	0.364	0.363
	*n*	6.011	1.676	16.484	5.141
	R^2^	0.998	0.999	0.926	0.744
	E^2^	2.00 × 10^−4^	2.00 × 10^−4^	1.10 × 10^−3^	0.61
Temkin	B_T_	2.91 × 10^5^	1.08 × 10^5^	1.43 × 10^5^	1.03 × 10^5^
	R^2^	0.988	0.92	0.878	0.907
	E^2^	1.00 × 10^−4^	1.00 × 10^−4^	8.00 × 10^−4^	1.50 × 10^−3^
	At	1.99 × 10^4^	32.73	1.0 × 10^9^	2.64 × 10^5^

**Table 4:** The parameters for pseudo-first-order and types 1–4 of pseudo-second-order.

Kinetics Type		Parameter	ChW		PTW	
			Cu^2+^	Pb^2+^	Cu^2+^	Pb^2+^
		*q*_e, exp_	0.080	0.046	0.137	0.145
Pseudo-first-order		*q*_e, cal_	0.068	0.1016	0.068	0.067
		k_1_	0.075	0.046	0.075	0.136
		R^2^	0.878	0.927	0.878	0.877
Pseudo-second-order	Type 1	*q*_e, cal_	**0.083**	**0.047**	0.141	0.149
		k_2_	1.152	6.518	0.750	0.772
		R^2^	**0.999**	**0.999**	0.999	0.999
		*h*	0.008	0.014	0.015	0.015
	Type 2	*q*_e, cal_	0.083	0.049	**0.139**	**0.144**
		k_2_	1.162	3.553	0.825	0.846
		R^2^	0.995	0.988	**0.988**	**0.934**
		*h*	0.008	0.008	0.016	0.018
	Type 3	*q*_e, cal_	10.500	5.878	8.873	8.957
		k_2_	1.153	3.495	0.810	0.763
		R^2^	0.995	0.988	0.988	0.934
		*h*	127.127	120.729	63.778	61.195
	Type 4	*q*_e, cal_	−0.028	−0.039	−0.036	3.602
		k_2_	5.351	7.569	3.740	3.602
		R^2^	0.982	0.979	0.954	0.890
		E^2^	0.004	0.011	0.015	0.005

Note: 1. The bold values are related with the parameters of the pseudo-second order kinetic model of type 1 which preferably describes the realized process of adsorption as compared to bold experimental data. 2. *h* is the initial adsorption rate, *h* = k_2_*q_e_*^2^.

**Table 5:** Linear forms of the pseudo-second-order kinetics model.

Type of Kinetics Pseudo-Second-Order	Linear Form	Plots
Type 1	$\frac{t}{q_{t}}=\frac{1}{k_{2}q_{e}^{2}}+\frac{t}{q_{e}}$	$\frac{t}{q_{t}}\;vs.\;t$
Type 2	$\frac{1}{q_{t}}=\left(\frac{1}{k_{2}q_{e}^{2}}\right)\frac{1}{t}+\frac{1}{q_{e}}$	$\frac{1}{q_{t}}\;vs.\;\frac{1}{t}$
Type 3	$\frac{1}{t}=\frac{k_{2}q_{e}^{2}}{q_{t}}-\frac{k_{2}q_{e}^{2}}{q_{e}}$	$\frac{1}{t}\;vs.\;\frac{1}{q_{t}}$
Type 4	$\frac{q_{t}}{t}=k_{2}q_{e}^{2}-\frac{k_{2}q_{e}^{2}}{q_{e}}$	$\frac{q_{t}}{t}\;vs.\;q_{t}$

**Table 6:** The intra-particle diffusion for ChW and PTW on Pb^2+^and Cu^2+^.

Samples	k_ind1_	C_1_	R_1_^2^	k_ind2_	C_1_	R_2_^2^	k_ind3_	C_3_	R_3_^2^
ChW-Cu^2+^	0.012	−0.002	0.9169	0.003	0.042	0.9643	0	0.077	1
ChW-Pb^2+^	0.011	−0.001	0.9495	0.002	0.032	0.9013	0	0.047	1
PTW-Cu^2+^	0.022	−0.004	0.9104	0.009	0.051	0.9911	0.001	0.118	0.8976
PTW-Pb^2+^	0.025	−0.003	0.9509	0.011	0.042	0.9863	0.001	0.126	0.7363
